# Will the Lord Lieutenant, the Bishop, or the Mayor of Worcester —One or All—Take Effective Action?

**Published:** 1913-08-23

**Authors:** 


### COUNTY HOSPITAL ADMINISTRATION AT WORCESTER.

### Will the Lord Lieutenant, the Bishop, or the Mayor of Worcester —One or All—Take Effective Action?

Great public interest has been excited by the leading article we published last week upon the Worcester Infirmary. The fullest possible confirmation and justification for our criticism and suggestions is the following report of the proceedings of the Infirmary Committee held on the 18th instant, which we take verbatim from the *Worcestershire Echo* of that date. Not one single fact, it will be seen, was controverted; not one single word of regret at the lamentable condition and state of affairs existing was uttered, and the only defence appeared to be that The Hospital, whose mission it is to help the hospitals and medical charities throughout the country, and to promote their efficiency, in virtue of its mission is described by some of the Committee as "the enemy." Certainly The Hospital is "the enemy" of incapacity, and of feeble and ruinous hospital administration wherever it exists. It is, however, the friend of efficiency and of the sick who are dependent upon the hospitals, of the medical profession, of the nurses and other hospital workers, who, in a case of lamentable breakdown like that at Worcester, must necessarily suffer when feebleness rules the roost. The *Worcester Daily Times* seems to refuse to judge the present management by its fruit. It indicates that several of the present Committee are old men, and wrongly assumes that our knowledge of the difficulties and their wise solution is incomplete. We judge from this and the drift of its Editor's article that he has not seen the various articles and suggestive recommendations we have published during the last few years. We have often urged how true it is that the inhabitants of a city like Worcester have the hospital which from their intelligence and energy, or the absence of these qualities, they deserve. Worcester must contain some good and able men of business, who we hope will come forward at this crisis and save its hospital with strong arms, keen brains, and willing hearts. Will not the Mayor, or the Bishop, or the Lord Lieutenant of the County put his hand to the plough? Each or all of them can at once provide that a strong committee of influential city and county residents shall be formed to save the Worcester Infirmary, and put it without delay upon a sound financial and administrative basis?

### "COMMITTEE RESENT 'HOSPITAL' ARTICLE."

#### Warm Discussion.

At to-day's (August 18) meeting of the Worcester Infirmary Committee there were present: Mr. G. Shrewsbury Smith (chairman), Revs. C. J. Hunt, R. N. Kane, Father Hart, Messrs. R. Bruce Ward, W. Park, H. A. Leicester, T. W. Byrne, S. T. Harris, T. A. Jarman, W. T. Curtler, J. G. Perry, T. Bates, sen., G. F. Adams.

Apologies for absence were sent by the Earl of Coventry, Revs. Canon Coventry and E. J. Boon, the Mayor of Worcester (Ald. E. Thomas), Messrs. J. Ward, T. P. Gostling, and H. B. Kenward.

Referring to a criticism of the management of the hospital in this month's Hospital, the Rev. R. N. Kane said although strongly objecting to it, they might take some lessons even from their enemies. Their great object was to increase their receipts, and they might take a hint from the criticism, although they did not like the general tenor of it. He suggested that the special sub-committee appointed should consider the proposals made in the article in The Hospital, and any other points that might arise.

Mr. Park said the sub-committee might review the whole question of increasing their income. He did not agree with a good deal of the strictures in the article. As regarded the remarks about incapacity, they could let these go.

Mr. Curtler protested against the strictures; they were uncalled for and objectionable. They were taking the matter lying down.

The Rev. C. J. Hunt suggested that they give notice that the committee did not acquiesce with the strictures, and considered them unjust. The indictment was against the whole work of the Infirmary for twenty years, and he proposed that they do not acquiesce with the implications with which the article abounded.

Ald. Leicester agreed with Mr. Hunt, and said the remarks in The Hospital were, in his judgment, made without proper knowledge of the facts—(hear, hear)—therefore they might ignore them. They might gather anything that might be useful in them and then do as they had been doing for some considerable time—try to put their house in order. They had re-appointed the committee to look after the reduction of the expenditure and the increase of income, and there was no doubt that much would come of it. But the first and most important thing was to give the citizens the opportunity, if they wished it, of learning the true modern history of their little difficulties. Then it would be for them to say whether they would do their duty or still go on grumbling. He would endeavour to write a short history of the present situation, which he would submit to the Chairman, and then send it to the Press. He was perfectly satisfied that when the intense ignorance of the citizens about the Infirmary was removed, they would see the dawn of better times. (Hear, hear.)

Mr. Harris suggested that they should not pass a resolution with regard to the article in The Hospital. They had already heard that they might learn from their enemies, and Mr. Leicester talked about "putting their house in order." They would be sure to get a reply more scathing than that one. He did not think it was wise to pass such a resolution. There was a lot in that article which he could endorse. To pass such a resolution would mean newspaper correspondence.

The Rev. C. J. Hunt said he was not particular about a motion being passed; he did not know the feeling of the committee. What he wanted, however, was that they should not take the criticism lying down. He admitted that there were things in it that might be considered. It was an extraordinarily scathing article, and he thought some protest should be made.

Mr. Park said they were condemned for things which they had considered.

This for a short time ended the discussion, members of the committee protesting against the criticism, but not passing any resolution.

Reply to Criticism Urged.

Mr. Adams re-opened the discussion later by asking if Mr. Leicester was going to send his letter to The Hospital —(A Voice: "Certainly not")—or whether it would be advisable to answer the letter at all.

Ald. Leicester explained, as stated, what he was going to do. He said he had been speaking to the Dean, and he grumbled, and other people grumbled, but they grumbled because in his (the speaker's) judgment they did not know the facts. He would treat The Hospital like he treated a good many of the papers.

Mr. Bates said it would be unwise to write a letter of protest to The Hospital, because they would be placing themselves in that paper's power. If the reply was a capable one they might not publish it, but if the opposite they would. That paper had put itself in a very wrong position indeed. If they sent any letter of protest, let it not be to that paper, because it had already tainted itself with the remarks, but to some other medical paper which would look at it in a fairer way.

Mr. Curtler agreed with Mr. Bates. The remarks were unjust and unlawful, because the writer was absolutely ignorant of the facts.

Mr. Adams said that without some reply the article would be taken as absolutely true. He did not suggest that a detailed answer be given, but a contradiction. It would be a great pity if it was not answered.

The Chairman was of opinion that it would be better to ignore the article altogether. They might—not that they were afraid—get into some newspaper war.

This closed the discussion.—*The Worcestershire Echo.*

